# Effects of salt stress on soil enzyme activities and rhizosphere microbial structure in salt-tolerant and -sensitive soybean

**DOI:** 10.1038/s41598-023-44266-5

**Published:** 2023-10-10

**Authors:** Dongwei Han, Di Zhang, Dezhi Han, Honglei Ren, Zhen Wang, Zhijia Zhu, Haoyue Sun, Lianxia Wang, Zhongcheng Qu, Wencheng Lu, Ming Yuan

**Affiliations:** 1 Qiqihar Branch of Heilongjiang Academy of Agricultural Sciences, Qiqihar, China; 2 Heihe Branch of Heilongjiang Academy of Agricultural Sciences, Heihe, China; 3 Soybean Research Institute, Heilongjiang Academy of Agriculture Sciences, Harbin, China

**Keywords:** Environmental microbiology, Salt

## Abstract

Salt is recognized as one of the most major factors that limits soybean yield in acidic soils. Soil enzyme activity and bacterial community have a critical function in improving the tolerance to soybean. Our aim was to assess the activities of soil enzyme, the structure of bacteria and their potential functions for salt resistance between Salt-tolerant (Salt-T) and -sensitive (Salt-S) soybean genotypes when subject to salt stress. Plant biomass, soil physicochemical properties, soil catalase, urease, sucrase, amylase, and acid phosphatase activities, and rhizosphere microbial characteristics were investigated in Salt-T and Salt-S soybean genotypes under salt stress with a pot experiment. Salt stress significantly decreased the soil enzyme activities and changed the rhizosphere microbial structure in a genotype-dependent manner. In addition, 46 ASVs which were enriched in the Salt-T geotype under the salt stress, such as ASV19 (*Alicyclobacillus*), ASV132 (*Tumebacillus*), ASV1760 (*Mycobacterium*) and ASV1357 (*Bacillus*), which may enhance the tolerance to soybean under salt stress. Moreover, the network structure of Salt-T soybean was simplified by salt stress, which may result in soil bacterial communities being susceptible to external factors. Salt stress altered the strength of soil enzyme activities and the assembly of microbial structure in Salt-T and Salt-S soybean genotypes. Na^+^, NO_3_^−^–N, NH_4_^+^–N and Olsen-P were the most important driving factors in the structure of bacterial community in both genotypes. Salt-T genotypes enriched several microorganisms that contributed to enhance salt tolerance in soybeans, such as *Alicyclobacillus, Tumebacillus*, and *Bacillus.* Nevertheless, the simplified network structure of salt-T genotype due to salt stress may render its bacterial community structure unstable and susceptible.

## Introduction

Soil salinization has seriously threatened agro-ecosystems, with approximately 30% of arable land affecting by salinization^1,2^. Soil salinization limits plant growth and thus leads to a reduction of crop production^1^. Due to the increase in global population and the ever-increasing demand for food quality, the need to reduce the pressure of salinity on saline soils, improve plant tolerance to salt stress and eventually increase crop yields is an urgent concern to be addressed. As one of the world's most important oil and protein crops, soybean is very sensitive to salt stress, which can severely restrict nutrient use and growth and development, ultimately reducing yields^3^. In the last few years, traditional breeding techniques combined with beneficial microorganisms have been widely used to improve the salt tolerance of soybeans^4,5^.

Root systems specific to different soybean cultivars determine different root exudates, which in turn shape the microbial community in the rhizosphere^6,7^. However, root exudates secreted by a variety of genotypes in the types and amounts were different under salt stress^2^. It has been demonstrated that salt-tolerant soybeans have a much greater salicin, phosphoglycolate, 1-methlseleno-N-acetyl-Dgalactosamine, arbutin 6-phosphate, and 5-O-3-phosphoshikimate than salt-sensitive soybeans in soils, which might increase the adaption of salt^2^. Different types and amounts of metabolites could alter the diversity and structure of rhizosphere microbes, which can assist the host to become more resistant to stress^2,8,9^. For instance, several potentially beneficial microorganisms were enriched in the rhizosphere, such as *Pseudomonas* have the ability to improve salinity stress in plants by producing stress-relieving metabolites such as exopolysaccharides, gibberellins, ACC deaminase and indoleacetic acid^10,11^. Thus, to understand how salt-resistant soybean better adapt to salt stress, it is necessary to investigate how rhizosphere microbes of salt-tolerant soybean genotypes respond to salt stress.

One of the important indicators of the metabolic dynamics of soil organisms is soil enzymes, which are mainly derived from plant secretions and microorganisms^7^. Furthermore, soil enzymes could mineralize nutrients for plants and micro-organisms and function in energy conversion and material cycling processes^12^. As enzyme activity varies with environmental factors, such as nutrient availability, the regulation of enzyme activities may be specific to soybean genotypes, which have an effect on the adaptation of soybean to salt stress^13^. For example, soil acid phosphatase was capable of releasing phosphate by hydrolyzing the phosphate ester bond of the phosphate group in organic molecules, and urease facilitates ammonium nitrogen (NH_4_^+^) release from urea, thereby alleviating salt stress in soybean^2^. Yet, the influence of genotype on enzyme activity in the soil under salt stress is still immature.

In this research, the enzymatic activity and bacterial structure of the rhizosphere under salt stress from salt-tolerant (Salt-T) and sensitive (Salt-S) soybean genotypes were measured. Real-time PCR and next-generation sequencing technologies were applied to detect the abundance and structure of bacterial community. We hypothesized that (1) Salt stress alters the soil enzyme activities in both genotypes of soybeans, with the Salt-T genotype exhibiting higher levels than the Salt-S genotype under salt stress, and (2) The Salt-T genotype has the ability to enrich certain salt-resistant microorganisms, thereby facilitating enhanced adaptation of soybeans to salt stress.

## Materials and methods

### Experimental design and soil sampling

In this study, soil used were collected in the farm land of Qiqihar (110°25′N, 21°32′E), Heilongjiang Province, China in June 2022. The soil chemical characteristics were: pH 7.8, porosity 48.2%, soil organic carbon (SOC) 8.5 g kg^−1^, total N 0.52 g kg^−1^, and total P 0.42 g kg^−1^. Two different soybean (*Glycine max L.*) genotypes were shown to be tolerant (Qinong7) or sensitive (Hefeng50) to salt stress. The pot experiment was conducted with six replicates per treatment in a greenhouse. A 4 mm mesh was used to sieve the soil. Ten seeds were sown for each pot and then two better seedlings were kept after the ninth day of sowing. The temperature range of the greenhouse was 16–20 °C at night and 25–30 °C in the daytime. Each treatment was watered with 150 mM NaCl solution, with equal amounts of pure water as a control. Soil moisture content was maintained at 85% of field capacity by calculating the weight of the pots on a daily basis.

All rhizosphere soil samples were collected at flowering stage (after 37d sowing), using shaking the roots. For each treatment, after 3 min shaking, a total of 5 g of soil were transferred into microcentrifuge tubes and then stored at -80 °C for DNA extraction. The remaining soils were kept at 4 °C to be analyzed for soil enzymes and soil properties.

### Soil physicochemical properties analysis

Soil physicochemical properties, including soil pH, TC, TN, TK, NH_4_^+^–N, NO_3_^−^–N, TP and Olsen-P were measured according to our previous study (Yuan et al., 2021). Moreover, the concentration of Na^+^ was determined with an atomic absorption spectrometry (AAS). According to the method previously described by Guan et al. (1986), the activities of sucrase acid phosphatase, amylase, catalase and urease were evaluated in this study.

### DNA extraction and quantitative real-time PCR (q-PCR)

Rhizosphere soil total DNA was extracted with a DNA SPIN Kit (MP Biomedicals, CA). q-PCR was conducted by the primers 341F (5′-CCTACGGGNGGCWGCAG-3′) and 805R (5′-GGACTACHVGGGTWTCTAAT -3′) for the bacterial abundance measurement^14,15^. The PCR reactions were conclude 20 µL PCR Supermix (Takara, Shanghai, China), 0.5 µL each primer (1 µM), and 10 ng DNA. The PCR condition were 97 °C for 10 min, and 35 cycles of 95 °C for 45 s, 60 °C for 40 s, and 75 °C for 50 s, and 75 °C extension for 1 min^16^.

### Microbial sequencing and bioinformatic analysis

For sequencing, primers 341F/805R were used to amplify the bacterial 16S rRNA gene V4 hypervariable region. The PCR amplification procedure was conducted within a reaction volume of 25 µl, comprising 20 µl of PCR SuperMix (Takara, Dalian, China), forward and reverse primers at a concentration of 20 µM each, and 10 ng of template DNA. The thermocycling conditions encompassed an initial step of denaturation at 94 °C for 50 s, succeeded by 29 cycles comprising denaturation at 96 °C for 30 s, annealing at 54 °C for 30 s, and elongation at 74 °C for 1 min. A final elongation cycle was performed at 74 °C for 8 min. Subsequently, the V4 amplicons were subjected to sequencing utilizing the Illumina MiSeq PE250 platform. The standard protocols were followed to paired-end sequence the pooled-purified in equimolar amounts of amplicons. All sequences in this study were deposited in the NCBI of the number PRJNA907417.

The raw sequencing data generated after sequencing were processed with QIIME Pipeline (version 1.19.1). In detail, based on the barcodes, each sample was assigned to the corresponding sequence using Cutadapt v.3.4 allowing one mismatch and low-quality sequences (average base quality score and the length less than 20 and 200 bp, respectively) were trimmed in VSEARCH. Chimeras in the sequence were then removed using UCHIME algorithm^17^. According to the best match to sequences in the RDP database, sequences were assigned phylogenetically by the RDP classifier^18^. Amplicon sequence variants (ASVs) were classified using CD-HIT at 97% sequence similarity and the α-diversity (Shannon diversity) was analyzed in QIIME^19^.

Principal co-ordinates analysis (PCoA), canonical correspondence analysis (CCA) and significance test (Adonis test and mantel test) were carried out in program R (version 4.0.2) using the package “vegan”. For identifying ASVs that were significantly associated with the separation of communities among genotypes, a generalized linear model with a negative binomial distribution was fitted to the normalized values of each of the 7,345 ASVs, and likelihood ratio tests were used to test for variations in abundances across different treatments. Venn analysis was conducted to show shared ASVs between treatments. SPSS v25.2 (IBM, USA) was used to check for correlation. Using the One-way ANOVA in Genstat 13, the differences in soil physiochemical properties and soil enzyme activities were evaluated.

### Network analysis

Association network analysis was applied to clarify the linkages that exist among the different microorganisms with a relative abundance of ASV > 0.1% in the bacterial communities of the two genotypes. Statistical significance was determined by Spearman's correlation coefficient more than 0.8 and *P*-value less than 0.05 between two ASVs^20^. In R (version 4.0.2), network topological characteristics were calculated to examine the relationships among bacteria. These characteristics included the count of positive and negative correlations, graph density, average clustering coefficient (avgCC), average path length (APL), network diameter, modularity (M), and average weighted degree (avgK). The results were visualized using Gephi^21^.

## Results

### Effects of salt stress on soybean biomass, soil physicochemical properties and enzyme activity

**Figure 1:**
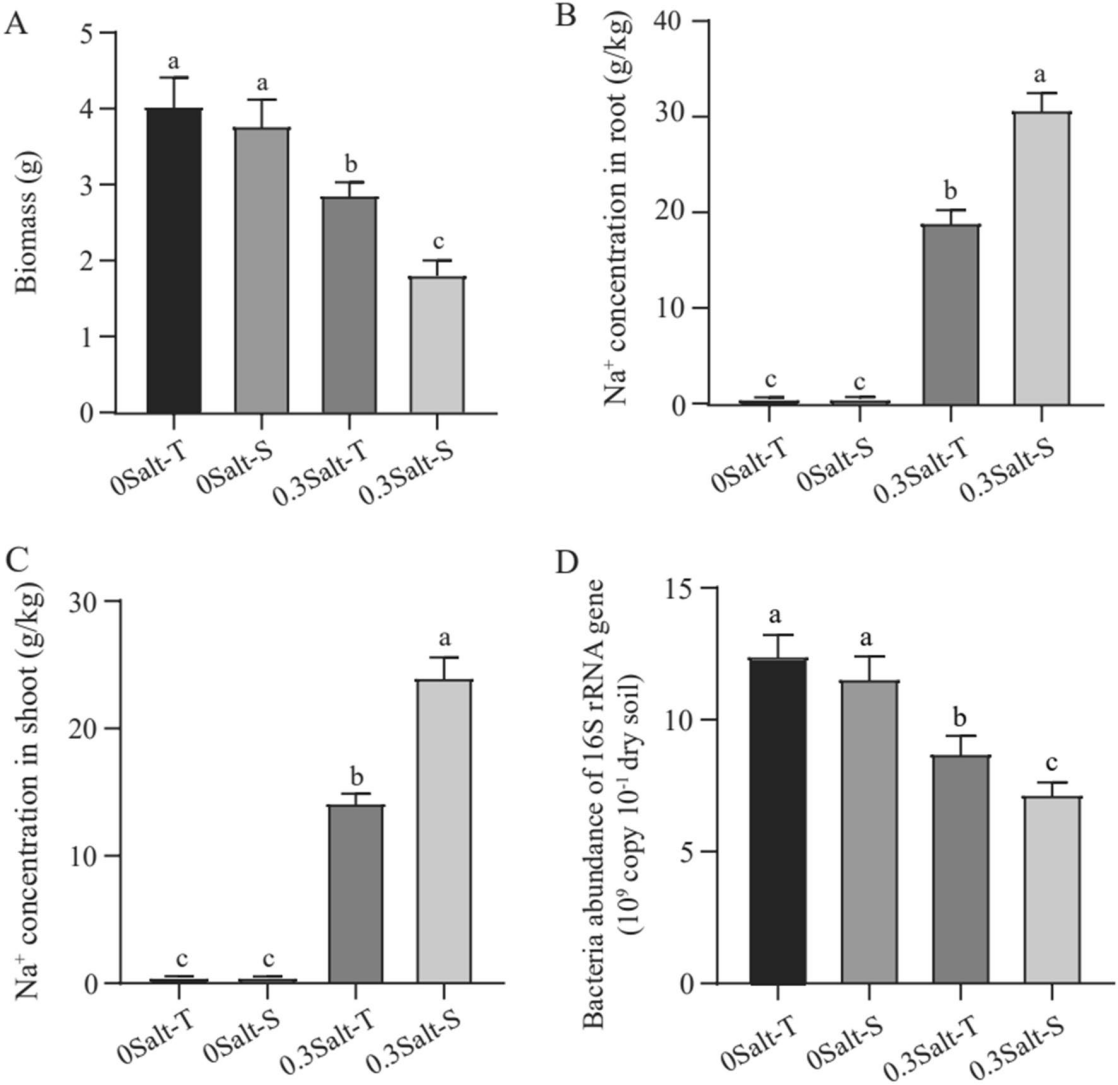
Plant biomass (**A**), concentrations of Na^+^ in roots (**B**) and shoots (**C**), bacteria abundance of 16S rRNA gene (**D**) respond to salt stress in Salt-T and Salt-S genotype.

Both genotypes were significantly reduced in biomass by salt stress. When the salt stress was applied, Salt-T genotype had 45% higher biomass than Salt-S genotype (*P* < 0.05), but not without salt stress (Fig. 1A). Moreover, salt stress led to higher levels of Na^+^ in roots and shoots, which were obviously increased in the Salt-S genotype than the Salt-T genotype (*P* < 0.05) (Fig. 1B,C).

Apart from acid phosphatase and amylase activities, significant differences were found in the soil enzymes activities among the two genotypes without salt stress. Yet, Salt-T showed significantly higher acid phosphatase, sucrase, catalase and urease activities than Salt-S soil under salt stress (*P* < 0.05) (Table 1). Specifically, the activity of urease in the Salt-T soil was 45% and 68% higher than in the Salt-S soil without and with salt stress, respectively. The value of acid phosphatase, catalase and sucrase activities were higher in the Salt-T than Salt-S under salt stress (Table 1). Additionally, urease activity was significantly positively correlated with NO3–N, Olsen-P and Na + in the Salt-S genotype, while not in the Salt-T genotype (Table 2). Moreover, acid phosphatase activity was significantly positively correlated with NO_3_^−^–N, NH_4_^+^–N, Na^+^ and pH in both genotypes (*P* < 0.05) (Table 2). Moreover, the two genotypes differed in the activities of catalases, sucrases, and amylases (Table 2).

**Table 1:** Enzyme activities respond to salt stress and genotypes.

Genotypes	Acid phosphatase	Sucrase	Urease	Amylase	Catalase
	(μg PNP g^−1^dw h^−1^)	(mol L^−1^ Na_2_S_2_O_3_ ml g^−1^)	(μg NH_4_^+^ g^−1^dw h^−1^)	(C_6_H_12_O_6_ mg g^−1^)	(mg O_2_ g^−1^dw s^−1^)
0Salt-T	41.42 ± 2.202 a	0.84 ± 0.162 a	0.16 ± 0.044 a	11.66 ± 1.187 a	3.51 ± 0.287 a
0Salt-S	40.56 ± 3.193 a	0.72 ± 0.045 b	0.10 ± 0.023 bc	10.68 ± 0.642 a	3.12 ± 0.224 b
0.3Salt-T	36.35 ± 2.031 b	0.47 ± 0.057 b	0.13 ± 0.023 ab	9.53 ± 0.555 b	3.00 ± 0.21 b
0.3Salt-S	33.09 ± 1.456 c	0.37 ± 0.072 c	0.067 ± 0.017 c	8.81 ± 1.14 b	2.42 ± 0.128 c
*P*	0.0001	0.0001	0.0001	0.0002	0.0001

Distinct lowercase letters were used to indicate significant differences between the treatments.

**Table 2:** Pearman's correlations between soil physicochemical properties and soil enzyme activities.

Genotypes		Acid phosphatase	Sucrase	Urease	Amylase	Catalase
Salt-T	C	0.389*	0.319*	− 0.204	0.405*	0.438**
	TN	− 0.151	− 0.057	− 0.017	− 0.116	0.206
	C: N	0.276	− 0.118	− 0.157	− 0.108	0.304
	TK	− 0.151	− 0.028	0.01	− 0.145	− 0.111
	TP	0.278*	− 0.13	− 0.128	0.188	0.198
	Olsen-P	0.151	0.467*	0.079	0.383*	0.131
	NH_4_^+^-N	0.635**	0.392*	− 0.031	0.301*	0.712***
	NO_3_–N	0.458**	0.697**	0.02	0.533**	0.485**
	Na^+^	0.345*	0.751**	− 0.001	0.456**	0.334*
	pH	0.393**	0.755***	0.061	0.495**	0.314*
Salt-S	C	− 0.081	− 0.045	0.249*	0.287	− 0.057
	TN	− 0.076	− 0.03	0.058	0.221	− 0.036
	C: N	0.031	− 0.01	0.009	0.089	− 0.101
	TK	− 0.076	− 0.021	0.276	0.138	0.053
	TP	− 0.197	− 0.068	− 0.001	0.101	− 0.053
	Olsen-P	0.611**	0.917***	0.255*	0.288*	0.813**
	NH_4_^+^-N	0.7**	0.716**	0.099	0.226	0.813***
	NO_3_–N	0.627***	0.79**	0.309**	0.419*	0.866**
	Na^+^	0.633**	0.793**	0.316**	0.526**	0.777**
	pH	0.691***	0.775**	0.227*	0.408**	0.84***

* *P* < 0.05; ** *P* < 0.01; *** *P* < 0.001.

### Soil bacterial abundance and diversity

**Figure 2:**
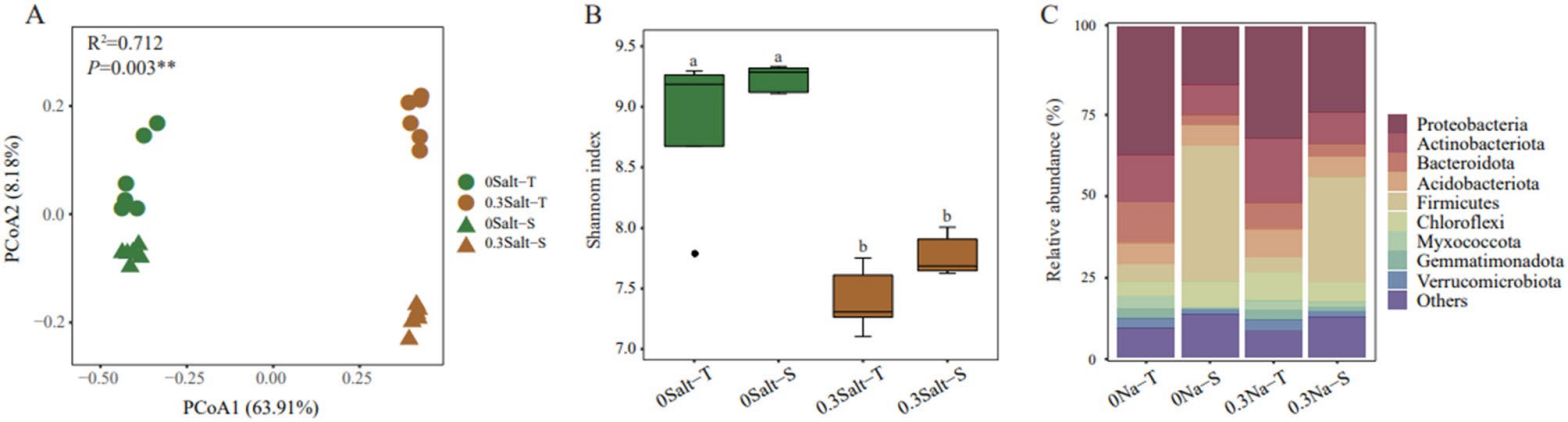
Effects of salt stress on Principal co-ordinates analysis (PCoA) (**A**), alpha diversity (**B**) and the relative abundances of the bacterial phylum (**C**).

The bacterial abundance for all soil samples ranged from 5.98 × 10^9^ to 12.67 × 10^9^ gene copies g^−1^ dry soil. In general, compared to the no salt condition, the soil bacterial abundance decreased with increasing salt concentration and was higher of Salt-T genotype (*P* < 0.05) (Fig. 1D). Shannon diversity was reduced by salt stress, with the Salt-T genotype decreasing even more (*P* < 0.05) (Fig. 2B). Overall, our results showed that salt stress significantly affected soil microbial abundance and diversity in both soybean genotypes.

### Soil bacterial community structure

PCoA showed significant separation for all soil samples along the first coordinate axis under the salt stress (*P* < 0.05) (Fig. 2A). The dominant bacterial phyla that had relative abundances of more than 5% in all soil samples, belonged to the Proteobacteria, Actinobacteria, Bacteroidota and Firmicutes. For all samples, their relative abundances ranged from 17.6% to 38.9%, 9.3% to 19.5%, 4.0% to 12.3%, and 4.5% to 41.0%, respectively (Fig. 2C). In addition, the phyla with low relative abundance, such as Chloroflexi, Myxococcota, Gemmatimonadota, and Verrucomicrobiota were also identified in the treatments (Fig. 2C). Generally, in term of the top four phyla, they could response to salt stress and differed among genotypes, which was in line with PCoA. In detail, Salt-T genotypes were significantly more abundant with Proteobacteria, Actinobacteriota and Bacteroidota (*P* < 0.05), while Salt-S genotypes had a higher relative abundance with Firmicutes phyla (*P* < 0.05) (Fig. 2C).

**Figure 3:**
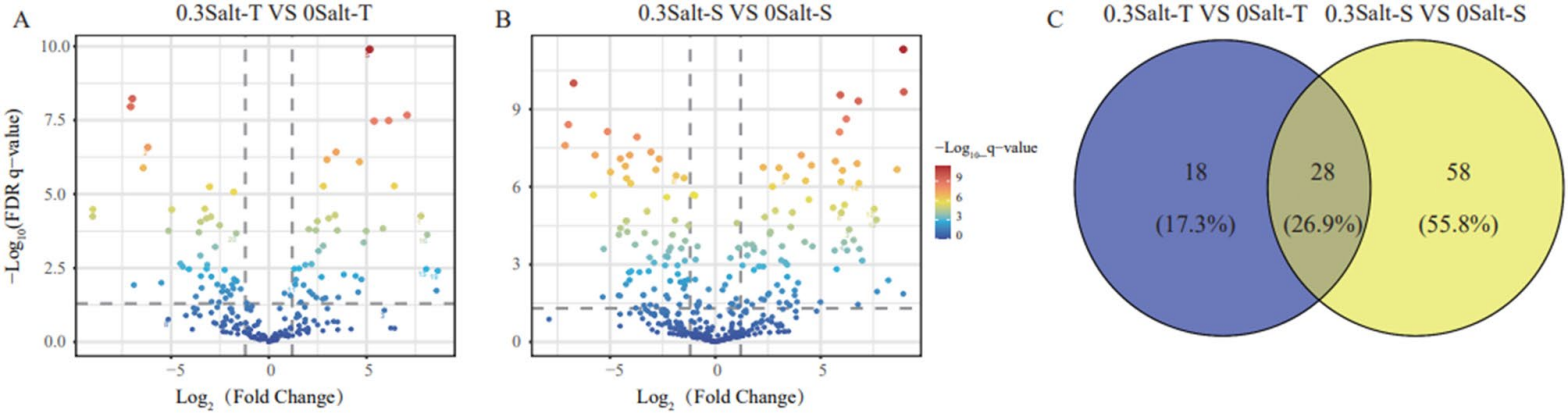
Differential abundance analysis shows the enriched and depleted ASVs of Salt-T (**A**) and Salt-S (**B**) included in the salt stress compared with the control. ASVs are represented by points, with their positions along the y-axis representing the abundance fold change in compared with control. Compared with control. The number of ASVs shared and unique between genotypes as shown in a Venn diagram (**C**).

By generalized linear model analysis, it was found that Salt-T genotype showed higher number of enriched and less depleted ASVs when compared to Salt -S genotype under salt stress (Fig. 3). There were 46 and 85 ASVs enriched in the Salt-T and Salt-S genotypes with salt stress, respectively (Fig. 3). It was noteworthy that 28 ASVs were co-enriched in the Salt-T and Salt-S genotype under the salt stress (Fig. 3, Table S2). Among them, many ASVs belonged to *Alicyclobacillus*, *Tumebacillus* and *Bacillus* (Table S2)*.*

### Effects of soil properties on bacterial communities

**Figure 4:**
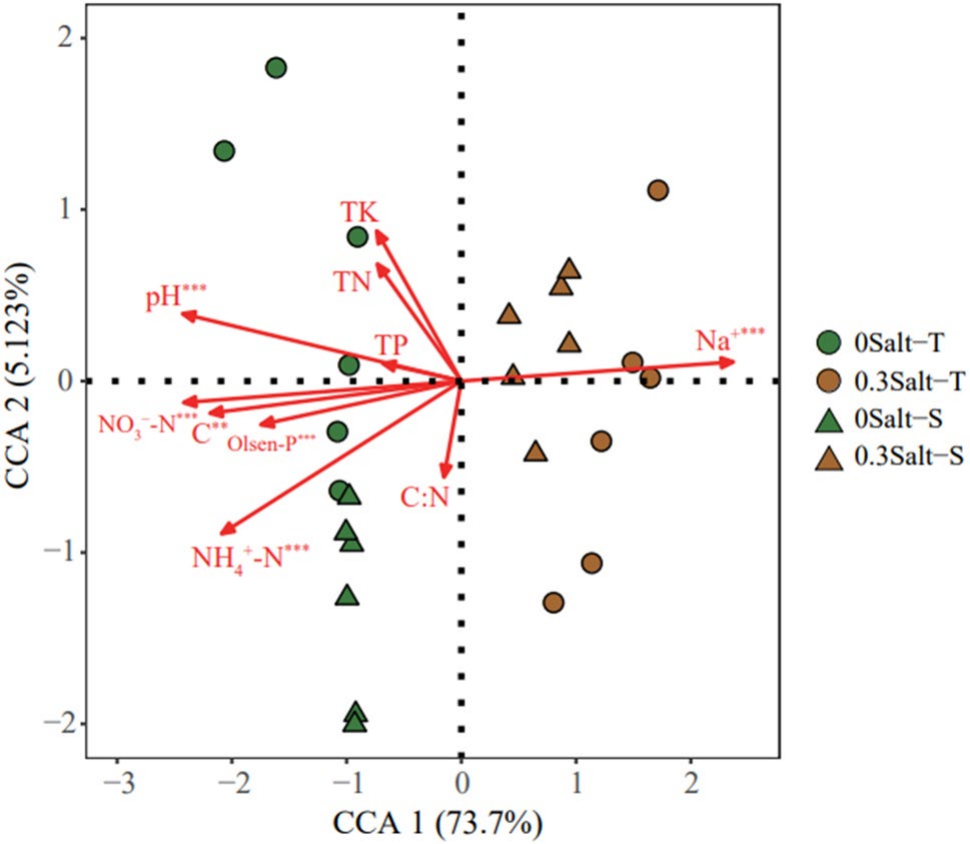
Canonical correspondence analysis (CCA) shows the relationship between bacterial communities with Salt-T genotype and Salt-S genotype and soil properties.

Canonical correspondence analysis (CCA) was conducted to build the linkages in soil chemical parameters and bacterial community composition (Fig. 4). Based on the Mantel test, Olsen-P, Na^+^, NH_4_^+^–N, pH, NO_3_^−^–N and C were determined to be associated with bacterial community structure for both genotypes (Fig. 4).

### Effects of salt stress on the association network

**Figure 5:**
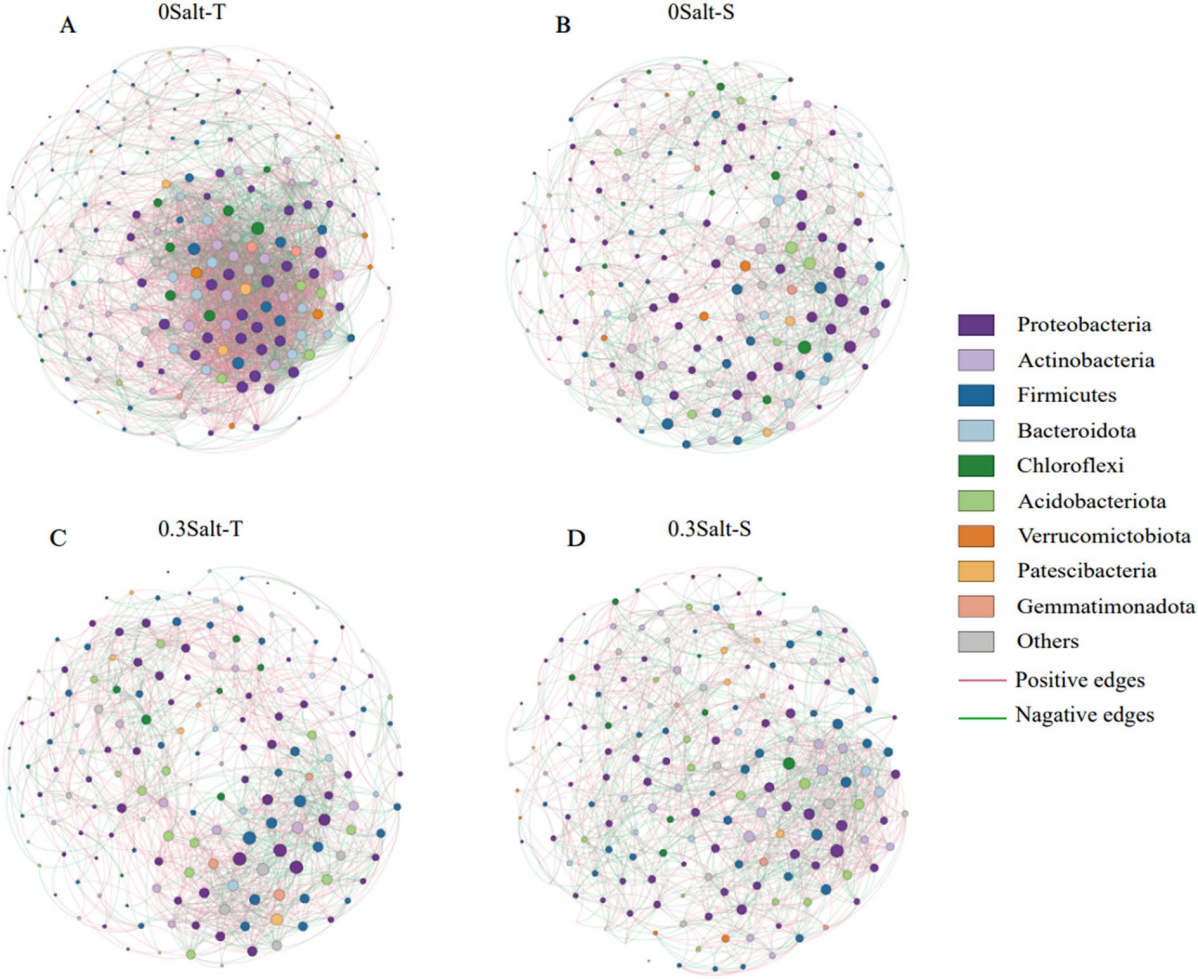
Network structure analysis of rhizosphere bacterial communities, e.g., 0Salt-T (**A**), 0Salt-S (**B**), 0.3Salt-T (**C**) and 0.3Salt -S (**D**). Different colors represent different phylum levels. The size of the nodes is positively related to the degree. Edge colors represent different correlations between two nodes.

As showed in the network analysis, the topologies of the network in two genotypes was significantly different by salt stress conditions (Fig. 5, Table 3). Specifically, the number of nodes and positive correlations and average weighted degree (avgK) decreased in Salt-T genotype under salt stress, while the opposite trend was observed in Salt-S genotype. Interestingly, modularity (M) increased in Salt-T genotype and decreased in Salt-S genotype. These results revealed that the network structure was made simpler in the Salt-T and more complicated in the Salt-S.

**Table 3:** Topological characteristics of the rhizosphere bacterial network of two genotypes with and without salt stress (0Salt-T, 0Salt-S, 0.3Salt-T and 0.3Salt -S).

Network Indicators	0Salt-T	0Salt-S	0.3Salt-T	0.3Salt-S
Modularity	0.913	5.373	2.499	4.031
Network Diameter	7	6	6	6
Average clustering coefficient	0.533	0.539	0.511	0.532
Number of nodes	196	177	171	181
Number of edges	3099	1480	1371	1547
Graph density	0.162	0.095	0.094	0.095
Average path length	2.643	2.943	3.015	2.893
Average weighted degree	15.153	3.911	6.556	4.648
Connecting components	5	1	4	2
Number of positive correlations	2009	877	881	931
Number of negative correlations	1090	603	490	616

## Discussion

This study aimed at unravelling for what alterations occur in rhizosphere soil enzyme activity and microbial structure in Salt-T and Salt-S soybean genotypes when exposed to salt stress. The initial hypothesis of our study was that Salt-T genotype exhibited higher soil enzyme activities than Salt-S genotype and might attract some salt-resistant microbes to enrich in the rhizosphere. Our results showed that salt stress significantly affected plant biomass, Na^+^ concentration in the plant, soil enzyme activity, the abundance and structure of bacterial and community both salt-T and salt-S genotypes. With these results, they are of great importance because soil enzymes and several bacterial genera are likely to exert key functions in enhancing soybean tolerance to salt stress.

### Salt stress affects the soil enzyme activity in soybean with different genotypes

Root secretions and microorganisms were two sources of enzyme production in the rhizosphere, respectively^7^. When exposed to stress, the root system usually secreted extra enzymes, varying by different genotypes^7^. Moreover, the increase in rhizosphere microorganisms led to an increase in the enzymes production to some extent^7,22^. In our study, urease, acid phosphatase, and catalase activities involved in N, P, and C cycling were remarkably stronger with the Salt-T genotype compared to the Salt-S genotype, which was associated with the increment of enzymes from the root system and higher bacterial abundance in the Salt-T genotype (Fig. 1)^23^.

In this study, the correlation between urease and acid phosphatase activities and soil physicochemical properties (e.g., Na^+^, NO_3_^−^–N, and Olsen-P) was higher in Salt- S genotype samples than in Salt- T genotype samples, indicating that soil enzyme activities of Salt-S genotype were more easily affected by salt stress (Table 2). There were antagonistic effects of salt on P absorption^24^. Acid phosphatase activity promoted the release of phosphate, thus helping soybean to counter low phosphorus stress and respond to salt stress^24^. However, a correlation was not found between acid phosphatase activity and Olsen-P in terms of Salt-T. This may be attributed to the stronger acid phosphatase activity in Salt-T genotypes and may enhance the release and decomposition of P related nutrients (organic phosphorus and Olsen-P), which mitigates P limitation^25,26^. Furthermore, some bacteria (e.g., *Burkholderia sp.* and *Enterobacter sp.*) that had relatively high abundance in the Salt-T genotype were capable of releasing Olsen-P in insoluble forms and fixed/adsorbed forms^27^. As a result of these facts lead to no significant connection between Olsen-P content and acid phosphatase activity in Salt-T genotypes.

We found a similar result indicating that Salt-T genotypes had higher bacterial abundance and lower Shannon diversity under salt stress when compared to those of the Salt-S genotype.The differential secretion of root exudates in response to stress conditions may explain the observed variations. Under salt stress, the Salt-T genotype exhibits higher expression of salt-tolerant genes, leading to the secretion of specific root exudates. These exudates may recruit beneficial bacteria that facilitate nutrient absorption in plants and regulate root sodium–potassium balance, thus aiding in salt tolerance^23,28^. Moreover, these genes can also regulate soybean root growth, which further influences the plant's response to salt stress. The combined effects of altered root exudates and regulated root growth contribute to the overall capacity of soybean plants to withstand salt stress^2^.

### Salt stress affects the bacterial structure in soybean with different genotypes

It has been accepted that plant genotypes and salt stress strongly affect soil bacteria community structures^2,29^. PCoA showed that salt stress altered the structure of bacterial community in both Salt-T and -S genotypes. It was in line with research by Lian^2^. They found different rhizosphere microbial communities in Salt-tolerant and -sensitive rice. Some functional genes involved in the biological processes of some metabolites in soybeans, such as *sst*, were associated with salt concentration level, especially in the salt-tolerant genotypes^2^. It would result in the release of more secondary metabolites such as citric acid, arbutin 6-phosphate, and salicin in salt-tolerant soybean, which would be more beneficial for the mitigation of salt stress^2,30^. On the other hand, these secondary metabolites had a strong ability to solubilize and chelate P, Fe, and Zn, which were more secretion in the Salt-T genotype to better improve the soil nutrient efficiency^30,31^. For instance, citric acid could promote the release of Olsen-P in the rhizosphere soil, which may be one of the factors leading to the shift of soil bacterial community under salt stress^28,32^.

By using differential ASV abundance analysis among treatments, the ASVs with higher relative abundance (Proteobacteria, Firmicutes and Actinobacteria) enriched in Salt-T soybean (Fig. 3 and Table S1) were observed. This was in line with the research by Lian^2^, which identified that some microbial species affiliated with the Proteobacteria and Firmicutes were with high relative abundance in the rice with tolerant to salt stress^2^. A significant finding of this study was that some Salt-T genotype ASVs had higher abundance in the Salt-T genotype than in the Salt-S genotype, such as ASV19 (*Alicyclobacillus*), ASV132 (*Tumebacillus*), ASV1760 (*Mycobacterium*) and ASV1357 (*Bacillus*) (Fig. 3, Table S1). This finding was in line with our hypothesis that salt-tolerant microorganisms would be activated by salt stress and thus played a crucial role in resisting the salt stress for Salt-T soybean. As study has reported that *Alicyclobacillus* was an iron-oxidizing bacteria and could grow in a low phosphorus environment, which might improve the solubilization of applied phosphates and fixed soil P^33,34^. *Alicyclobacillus* had a high abundance and might elevate the tolerance of soybean in the rhizosphere. Moreover, *Tumebacillus* had the ability to produce amylase, a key enzyme engaged in the carbon cycle that hydrolyzed starch into maltose and dextrin, providing microorganisms with nutrition^35^.

### Salt stress alters the bacterial association network in soybean with different genotypes

To develop a deeper knowledge of bacterial community composition, we conducted the association network analysis to compare the network complexity of two genotypes of soybean^21^. Salt stress had significant influences on the bacterial networks of different genotypes. In Salt-T genotype, several topological features such as the graph density, the number of edges and positive correlations, average clustering coefficient and average degree decreased with increasing salt stress, implying a simplified network in Salt-T genotype, while the Salt-S genotype was opposite to Salt-T (Fig. 5, Table 3). A possible explanation is that the enrichment of several specific microbial taxa by the Salt-T soybean in order to adapt salt stress, which cause the microbial community structure to move out of its inherent equilibrium state. Moreover, the higher average degree (avgK) and modularity (M) in the Salt-S soybeans suggested that possibly more coupling, exchange, and cooperation were found in the dominant microbes. In summary, all of these results indicated that salt stress simplified the salt-T soybean network, and this might render the microorganisms in the soil sensitive to external conditions^36^.

In summary, this study shows that bacterial structure and enzyme activity in rhizosphere soils respond differently to salt stress in salt-T and salt-S soybean genotypes. Salt-T soybeans could be enriched with some salt-tolerant microbes like *Alicyclobacillus, Tumebacillus*, *Mycobacterium* and *Bacillus*, thus enhancing the tolerance to salt stress. However, *Mycobacterium* have not been reported as salt-tolerant bacteria, therefore, the mechanism of this genus for plant response to salt stress is worth further study. Moreover, the network structure of Salt-T soybeans was simplified when subjected to salt stress, which might render the soil bacterial community sensitive to other stresses. However, the function of these salt-tolerant microorganisms is needed to explore by further experiments, such as transplanting them from tolerant plants to sensitive plants. Overall, this study provides insights into the responses of soil enzymes, bacterial communities, and their potential contributions to salt tolerance in soybean genotypes. The findings contribute to our understanding of the complex interactions between plants and microbes under salt stress conditions and highlight the importance of microbial-mediated processes in improving salt tolerance in soybean cultivation.

### Supplementary Information

Supplementary Tables.

## Data Availability

All sequences in this study were deposited in the NCBI of the number PRJNA907417.
